# Simultaneous robotic subtotal gastrectomy and right hemicolectomy for synchronous adenocarcinoma of stomach and colon

**DOI:** 10.1007/s11701-017-0681-5

**Published:** 2017-01-21

**Authors:** Byoung Jo Suh, Sung Jin Oh, Jin Yong Shin, Do Hoon Ku, Dong Sik Bae, Jong Kwon Park

**Affiliations:** Department of Surgery, Haeundae Paik Hospital, Inje University College of Medicine, 875 Haeundaero, Haeundae-gu, Busan, 612-862 Korea

**Keywords:** Gastric cancer, Colon cancer, Robotic surgery

## Abstract

Simultaneous laparoscopy-assisted resection for synchronous stomach and colon cancers has been reported frequently; however, robot-assisted gastrectomy and colectomy for these conditions are rarely reported. We report the successful use of robotic surgery for synchronous cancers of the stomach and colon. A 71-year-old woman with no specific medical history was diagnosed with early gastric cancer at the gastric angle and right colon cancer after undergoing esophagogastroduodenoscopy and colonofiberoscopy. Abdomino-pelvic computed tomography revealed that the stomach and colon lesions were limited to the mucosa without any lymph nodes or distant metastasis, which suggested the clinical stage for both cancers as T1N0M0. We performed robot-assisted radical subtotal gastrectomy and simultaneous right hemicolectomy through six ports. All procedures were successful without any perioperative complications. A 36-month postoperative follow-up of the patient at the outpatient department revealed no evidence of recurrence. We consider that concurrent robot-assisted subtotal gastrectomy and colectomy are technically feasible and safe.

## Introduction

Since the first robotic cholecystectomy performed by Cardiere [1], robotic surgeries have been applied widely in many specialties. The first robotic colectomy and gastrectomy were reported by Weber et al. [2] and by Hashizume et al., respectively [3]. It has been reported that the most common synchronous neoplasm associated with gastric cancer is colorectal cancer with the range of incidence from 0.8 to 3.9% [4, 5]. Open procedure for synchronous gastrectomy and colectomy is necessary for making a long incision from the upper abdomen to the lower abdomen. Recently, many studies have been conducted regarding concurrent laparoscopic surgery for stomach and colon cancers [6, 7]. However, concurrent robotic surgery for synchronous stomach and colon cancers is very rarely reported. We report a successful concurrent robot-assisted surgery performed on a 71-year-old woman with early gastric cancer (EGC) and colon cancer.

## Case reports

**Fig. 1:**
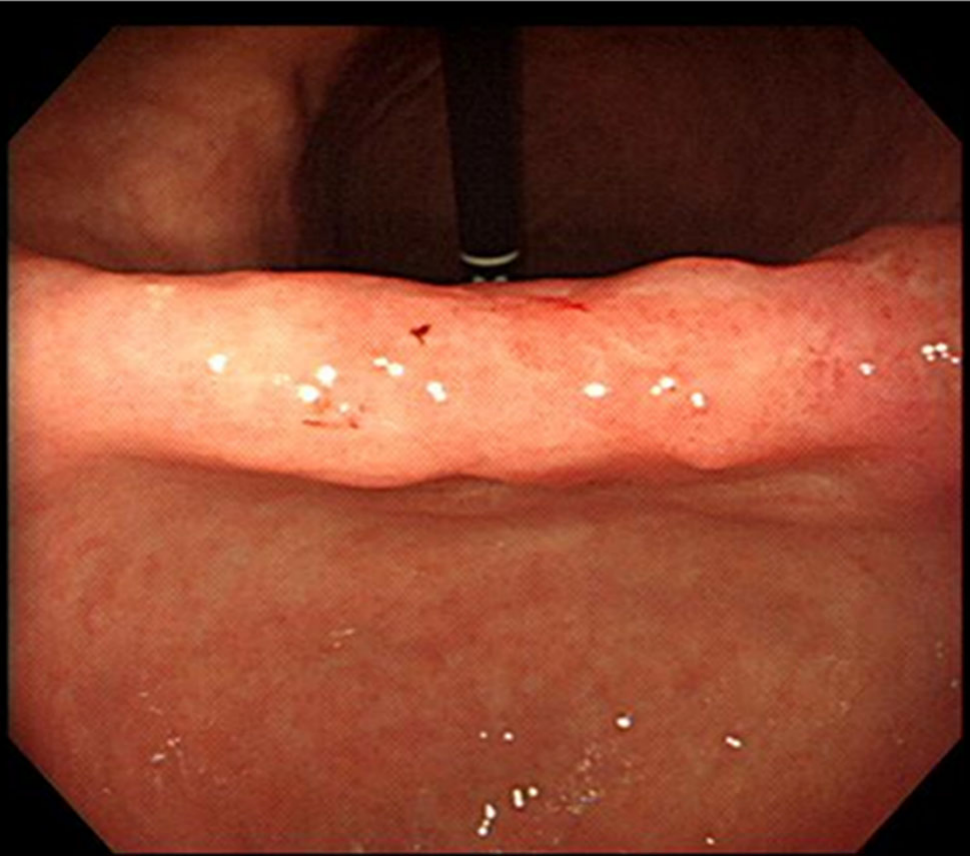
EGC IIa lesion at the angle of the stomach that was diagnosed as signet ring cell carcinoma

**Fig. 2:**
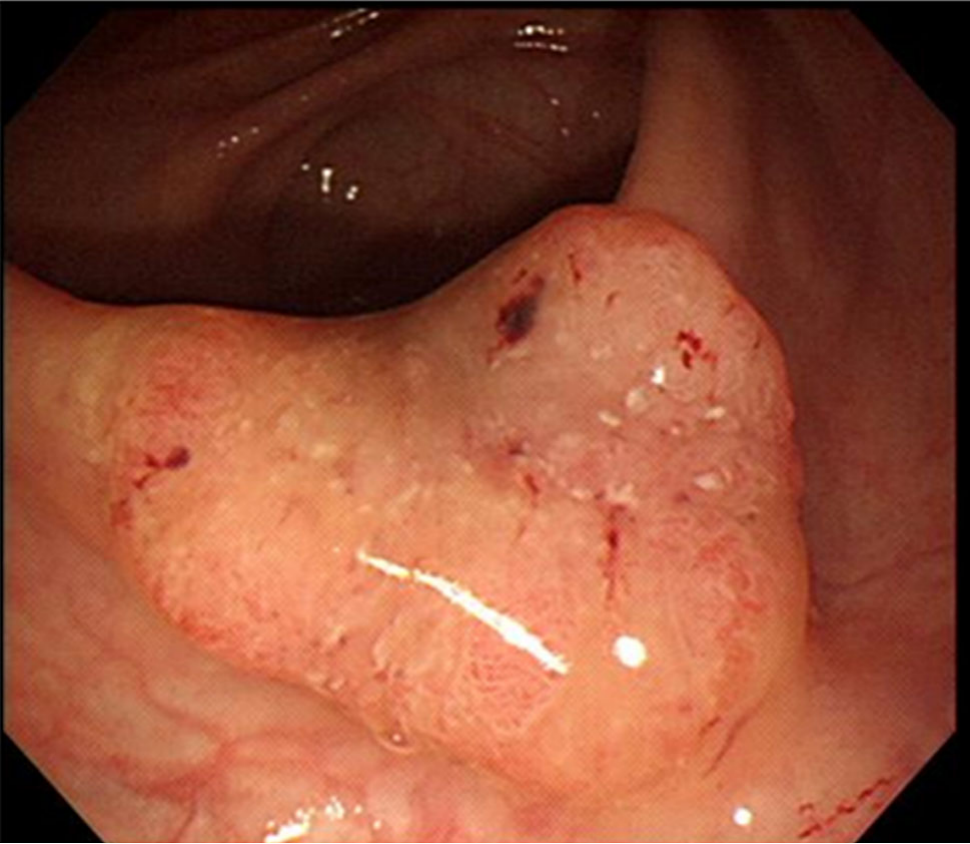
Elevated lesion at the cecum that was diagnosed as a well-differentiated adenocarcinoma

A 71-year-old woman with no specific medical history was diagnosed with EGC and right colon cancer after undergoing esophagogastroduodenoscopy (EGD) and colonofiberoscopy (CFS). Abdomino-pelvic computed tomography (CT) revealed that gastric and colon cancers were limited to the mucosa without any lymph nodes or distant metastasis, which suggested the clinical stage for both cancers as T1N0M0. Routine laboratory test results were normal. On physical examination of the patient after admission, her body weight, height, and hence the preoperative body mass index (BMI) were observed as 51 kg, 159 cm, and 20.17 kg m^2^, respectively. EGD revealed an EGC IIa lesion at a gastric angle, and CFS revealed an elevated lesion at the cecum, thus indicating signet ring cell carcinoma (Fig. 1) and well-differentiated adenocarcinoma (Fig. 2), respectively. Based on these findings, the patient was diagnosed with synchronous stomach and right colon cancers. We decided to perform robot-assisted distal gastrectomy (RADG) and right hemicolectomy simultaneously after obtaining an informed consent for the procedure. On April 4, 2011, RADG with D1+β lymph node dissection and Billroth II anastomosis were performed. After performing gastrectomy, robot-assisted right colectomy was performed by the team of colorectal surgeons.

## Surgical procedures

### Robot-assisted radical subtotal gastrectomy

**Fig. 3:**
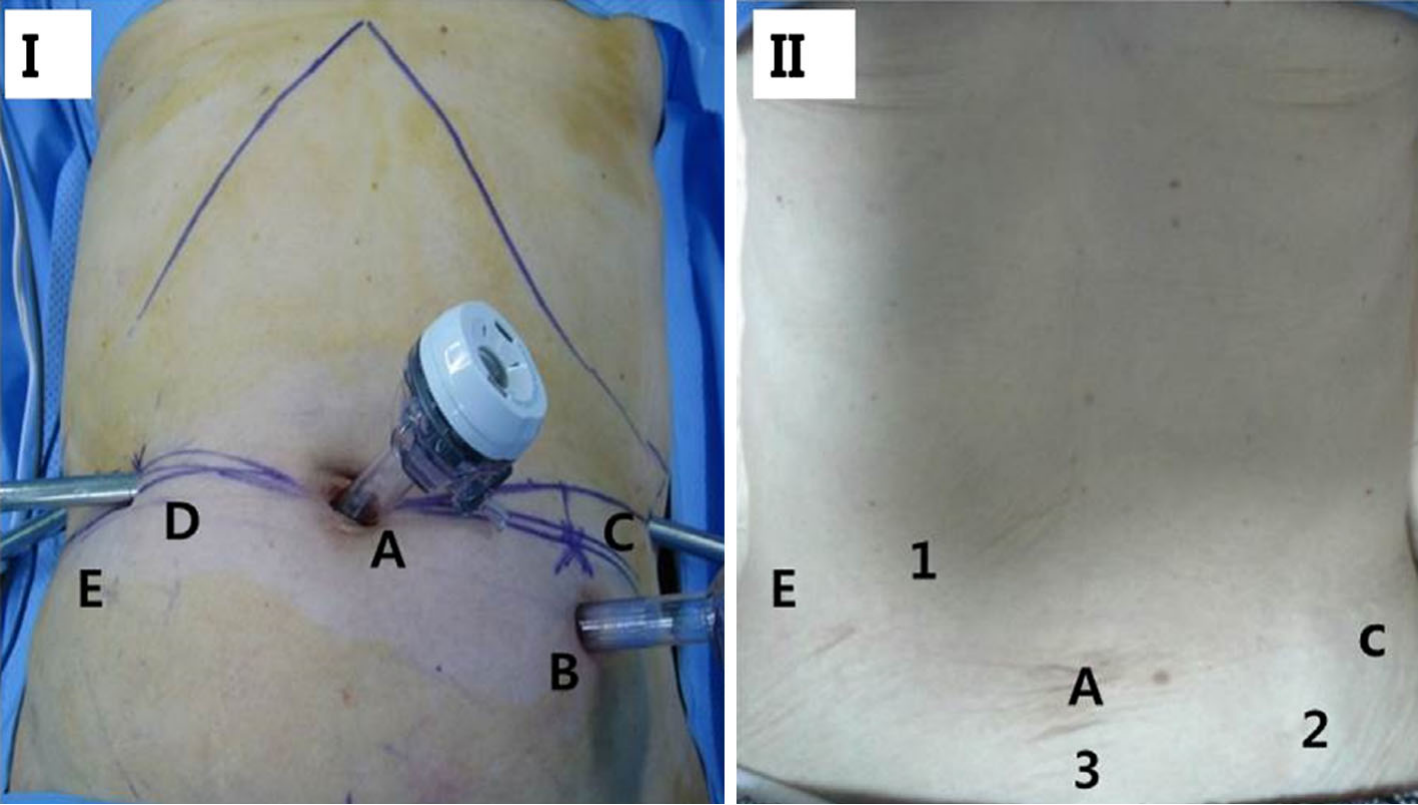
**I** Port placement. Robotic gastrectomy. *A* camera port, *B* assistant port, *C* 1st robot arm port: Maryland Bipolar Forceps, *D* 2nd robot arm port: Harmonic Curved Shears, *E* 3rd robot arm port: Cadiere Forceps. Robotic colectomy: *A* camera port *1* 3rd robot arm port: double fenestrated grasper, *2* 1st robot arm port: hot shears, *3* suprapubic: 2nd robot arm port: Bipolar Grasper Forceps. **II** Abdominal surgical wound (POD 36 months)

We inserted five ports (Fig. 3). The first 12-mm trocar was inserted into the infraumbilical area using the open method (open Hassan technique) and a pneumoperitoneum was created; further, intracorporeal pressure was increased up to 12 mm Hg by CO_2_ gas. A dual lens scope was inserted through this trocar. Additionally, four trocars were placed under direct visualization. Two 8-mm trocars were placed on the patient’s right side and one 8-mm trocar was placed on the patient’s upper left side. A 12-mm trocar was placed on the patient’s central left side for assistance. After docking the robotic arms with trocars, Cadiere forceps (Intuitive Surgical Inc., Sunnyvale, CA, USA) were inserted through the right upper 8-mm trocar, and a pair of ultrasonic coagulating shears (Harmonic scalpel, Ethicon Endosurgery Inc., Cincinnati, OH, USA) was inserted through the right central 8-mm trocar. Maryland bipolar forceps (Intuitive Surgical Inc., Sunnyvale, CA, USA) were inserted through the left outer 8-mm trocar, and initially, the liver was retracted using Prolene with two stylets being inserted into its lateral segment before docking the robotic arms. The greater omentum was resected using ultrasonic coagulating shears (Harmonic scalpel, Ethicon Endosurgery, Inc., Cincinnati, OH). After dividing and ligating the left gastroepiploic vessels at their roots, dissection around the lymph nodes was performed toward the pylorus. Further, the right gastroepiploic vessels were divided and ligated at the roots. After ligating the right gastric artery, the duodenum was transected 1–2 cm distal to the pyloric ring using a linear stapler, Echelon blue (Ethicon Endosurgery, Inc., Cincinnati, OH) device. After performing gastrectomy at the surgeon consoles, the robot was separated from the patient. A 5-cm-long minilaparotomy was made at the upper abdomen, a wound protector (Alexis TM Wound retractor, Applied Medical, Rancho Santa Margarita, CA) was applied to the laparotomy site, and the stomach was resected.

### Robot-assisted radical right hemicolectomy

After applying glove to the Alexis and creating the pneumoperitoneum with CO_2_ gas, we inserted only one additional 8-mm port at the suprapubic area for the use of bipolar grasper forceps (Fig. 3). Further, after redocking of the da Vinci system, a 12-mm assistant port that was used for stomach cancer operation was used for the first robotic arm to insert the bipolar scissors. Additionally, the right midclavicular 8-mm port for harmonic scalpel use during stomach cancer operation was changed to the third robot arm port for use of double fenestrated grasper in robotic right colectomy. Because the target organ was changed from the stomach to the right colon, the da Vinci system was introduced from the patient’s right side. First, traction was applied to the mesentery of the terminal ileum with the third robotic arm. Mobilization of the colon was carried out inferior to the superior direction in the avascular plane between Gerota’s fascia and Toldt’s fascia. During this procedure, the duodenum was used as a landmark for safe upward dissection. The ileocolic vessel was then isolated and separately ligated near the superior mesenteric vessel with robotic clips (Hemolock). Thereafter, by performing a dissection along the lateral part of the middle colic vessel, the middle colic vessel was severed. The gastrocolic trunk from the superior mesenteric vein was then exposed and its branch was ligated with a robotic clip. The greater omentum was dissected toward the hepatic flexure. After completing the dissection, the specimen of the right colon was carried out through the previously made epigastric minilaparotomy, and ileocolostomy was performed extracorporeally. Next, we performed extracorporeal Billroth II anastomosis, and all the procedures were completed without inserting a nasogastric tube and by inserting one closed suction drain. The total operation time was 640 min. For the robotic gastrectomy, the docking time was 5 min and the console time was 300 min. For the robotic right hemicolectomy, the docking time was 10 min and the console time was 100 min. Bleeding amount was observed as 420 cc without intraoperative complications. The postoperative course was uneventful. Flatus occurred on the third postoperative day and commencement of oral feeding with water was permitted on the fourth postoperative day. On the fifth postoperative day, the patient was allowed to take liquid diet, and soft diet was allowed from the sixth postoperative day. The patient was discharged on the 11th postoperative day. The final pathologic findings confirmed mixed signet ring cell carcinoma and tubular adenocarcinoma, poorly differentiated, diffuse type on Lauren with depth; the mucosa; and 0 of 20 retrieved lymph nodes (pT1N0M0) in the case of stomach cancer. For the colon cancer, pathologic findings confirmed well-differentiated adenocarcinoma at the ascending colon, with depth of invasion, and muscularis propria without lymph node metastasis in the 14 retrieved regional lymph nodes (pT2N0M0). We followed up the patient at the outpatient department for 36 months after the operation and no problem was observed.

## Discussion

We report a simultaneous robotic curative surgery for synchronous early gastric cancer and early cecal cancer. There have been many reports concerning laparoscopic combined resection in patients with synchronous gastric and colorectal cancers that include its safety and technical feasibility [5–8].

However, concurrent robotic surgery for the synchronous gastric and colon cancers is a very rare procedure. To the best of our knowledge, there are no reports of simultaneous robotic curative resection for synchronous gastric and colon cancers.

Surgical resection with lymphadenectomy is the gold standard treatment for both gastric and colorectal cancers. Minimally invasive surgery (MIS) is widely accepted in the field of gastric and colorectal cancers. In terms of surgical trauma and postoperative quality of life, laparoscopic surgery demonstrated its superiority to the open surgery [6, 7]. Minimal postoperative inflammation and small surgical wounds enable the patient to recover earlier and obtain better cosmetic results than the conventional open surgery.

Synchronous gastric and colon cancer surgery with conventional open procedure should be performed with a long midline incision from the xiphoid process to the lower abdomen because of the different anatomic location of the two organs. However, minimal invasive surgical procedures such as laparoscopic or robotic surgery have advantages involving exploration of a larger area of multiquadrant abdominal organs with better visualization for small surgical wounds [8].

Robotic surgery has overcome the limitations of laparoscopic approach. The advantages of robotic surgery include ten times magnified three-dimensional surgical view and articulated motion of surgical instruments. These might be more helpful to perform precise lymph nodes dissection [9].

Concerning the technical aspects of robotic simultaneous procedures of synchronous double cancers, it is very important to appropriately place ports to maximize the advantage of minimally invasive procedures. We added only one additional port for colectomy after completing gastrectomy. Because robotic arms are tough and large, with an additional port for performing robotic right colectomy, we experienced some bumping of the robotic arms. Therefore, it is important to carefully insert the additional port to minimize the collision of the robotic arms.

Robotic surgery takes longer than conventional open or laparoscopic surgery [10]. This was also observed for simultaneous operations for synchronous cancers. Despite the longer operation time, the postoperative outcome of our case was acceptable: first, flatus and oral resumption were quickly achieved without any complications. However, the total operation time was 640 min, which is longer than that with laparoscopic and open approaches. According to some researchers, patients with gastric cancer may be at an increased risk of developing synchronous or metachronous colorectal cancer. In several Asian countries, including Korea and Japan, the frequency of synchronous gastric and colorectal cancer ranges between 0.8 and 4%. Saito et al. reported that when colonoscopy was selected as the screening method, synchronous colorectal cancer was detected with a high prevalence at 4% [10]. Therefore, they recommended performing a screening colonoscopy before the treatment if possible for patients with gastric cancer. Because colorectal cancer is the most commonly diagnosed synchronous cancer and the incidence of colorectal cancer is increasing rapidly in Korea [11], we recommend colonofiberoscopy in elderly patients who are diagnosed with stomach cancer before the surgery.

To our knowledge, few reports on synchronous robot-assisted resection with regional lymph node dissection for gastric and colon double cancer have been published. We conclude that robotic surgery of synchronous stomach and colon cancers is a technically feasible and safe procedure.
